# Unilateral single-port thoracoscopic surgery for bilateral pneumothorax or pulmonary bullae

**DOI:** 10.1186/s13019-019-0894-y

**Published:** 2019-04-11

**Authors:** Xiaojian Li, Xiaojin Wang, Huayong Zhang, Hua Cheng, Qingdong Cao

**Affiliations:** The Fifth Affiliated Hospital of Sun Yat-sen University, Zhuhai, 519000 Guangdong China

**Keywords:** Tubeless, single-port, video-assisted thoracic surgery, mediastinum, pneumothorax

## Abstract

**Background:**

Rapid rehabilitation surgery has become a widely accepted approach. Thoracic surgeons have attempted in many ways to make surgery less invasive. We combined tubeless technology, single-port technology and mediastinum approach for the treatment of simultaneous bilateral primary spontaneous pneumothorax(PSP)or pulmonary bullae. And we evaluated its therapeutic effect. This study aimed to investigate if tubeless single-port video-assisted thoracic surgery (Tubeless-SPVATS) via anterior mediastinum can be used as an alternative surgical treatment for bilateral lung diseases, especially for concurrent or contralateral recurrence PSP.

**Methods:**

From November 2014 to December 2016, 18 patients with simultaneous bilateral PSP or pulmonary bullae were treated with tubeless -SPVATS via anterior mediastinum. They were 13 males and 5 females with an average age of 20.2 ± 2.3 years (17 to 24 years). They all had preoperative chest CT and were diagnosed with simultaneous bilateral PSP or pulmonary bullae.

**Results:**

Fifteen patients underwent bilateral bullae resection with Tubeless-SPVATS via anterior mediastinum. Three patients underwent bilateral single-port video-assisted thoracic surgery. No thoracotomy was performed. No death and grade 3-4 mobidity were found. All the patients started eating 6 hours after surgery. The average operation time was 44.56±17.8min. The patients were discharged 3. 5±1.0 days postoperatively.

**Conclusions:**

Tubeless-SPVATS via anterior mediastinum is a safe and feasible treatment for patients with simultaneous bilateral PSP or pulmonary bullae. However,contralateral thoracic is not explored fully enough. And when contralateral lung bullae are located near the hilum, endoscopic linear stapler cannot be easily used to conduct suture. Thus, the recurrence rate after performing Tubeless-SPVATS may be increased compared to performing thoracotomy. However, compared to bilateral thoracic surgery, this method reduced postoperative pain. And it took significantly less time than bilateral thoracic surgery. Thus, this method has some clinic value.

## Introduction

Thoracic surgery has long been one of the main ways to treat PSP. Recently, rapid rehabilitation surgery has become a widely accepted approach. Thoracic surgeons have attempted in many ways to make surgery less invasive. We have accumulated some experience, since 2011 when we reported single-port video-assisted thoracic surgery in China. This study aimed to investigate if tubeless single-port video-assisted thoracic surgery (Tubeless-SPVATS) via anterior mediastinal approach can be used as an alternative surgical treatment for bilateral lung diseases, especially for concurrent or contralateral recurrence PSP.

## Methods

### Information

From November 2014 to December 2016, 18 patients with simultaneous bilateral PSP or pulmonary bullae were treated with tubeless single-port video-assisted thoracic surgery (Tubeless-SPVATS) via anterior mediastinum. They were 13 males and 5 females with an average age of 20.2 ± 2.3 years (17 to 24 years).The characteristics of the patients are shown in Table 1. All of them recovered within one week. There was no history of thoracic surgery and no history of lung disease. They all had preoperative chest CT and were diagnosed with simultaneous bilateral PSP or pulmonary bullae. Preoperative chest CT showed no obvious chest adhesions. It also showed if there were any pulmonary bullae. If there was any, it also showed the location of them. We fully informed the patients and their guardians about the benefits and risks of this approach preoperatively.

**Table 1:** Clinical characteristics for all patients

Characteristics	All patients
Age (years)^a^	20.2 ± 2.3
Sex (male:female)	13:5
BMI※ (kg/m2)^a^	19.5± 2.5
Smoking(yes:no)	8:10
Pneumothorax size^a,b^	0.5±0.33
Average operation time(min)^a^	44.56±17.8
Intraoperative blood loss(ml)^a^	20±7.5
Postoperative hospital stay^a^(day)	3. 5±1.0

^a^Values are the mean±standard deviation for continuous variable. ※BMI = body mass index. ^b^Estimated by Light index

**Fig. 1:**
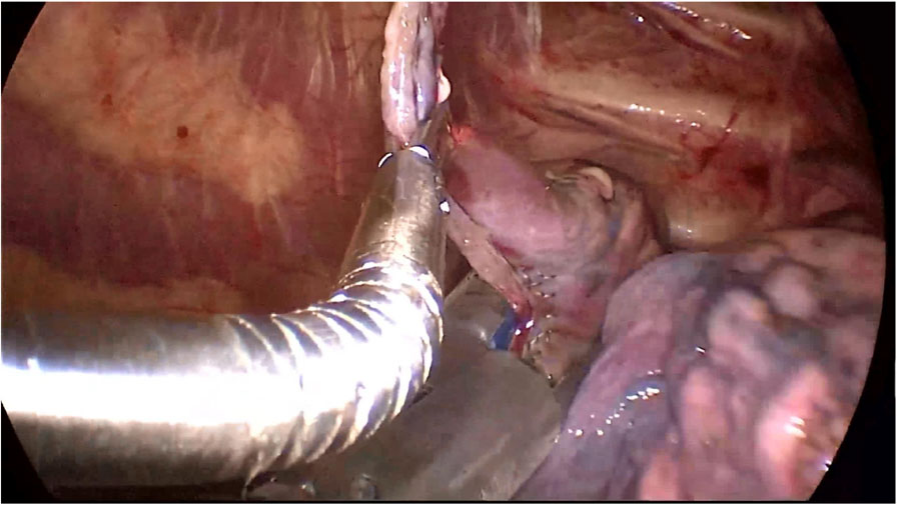
Wedge resection was used to resect the bullae

### Equipment

Independent research and development of double-joint single-port thoracic surgical instruments [1]. The features of the instrument included dendable double joint instrument, bionic design, and capable of bending to 7 different degrees.

A 30° and 5 mm thoracoscope (KARL STORZ, Germany) was used in this surgery.

### Operative procedure

#### Anesthesia and body position

- Anesthesia: Non - tracheal intubation autonomic respiratory mask anesthesia combined with epidural anesthesia
- Body position: The patient was recumbent with semi-supine position and double upper extremity abduction 90° fixed.

#### Surgeon and assistant station

The surgeon was on the side of the surgery, which was convenient to operate. The person who held the thoracoscope was located on the same side of the surgeon.

#### The incision principles

According to preoperative CT scan, we decided to make the incision from the complex and hard to operate side. The incision typically placed in the fifth or sixth intercostal axillary to axillary line. The location is easy to probe the entire chest and contralateral chest, especially the costophrenic angle, lung bottom and other areas. The rib gap here is relatively wide, easy to operate and there is less incision harassment. Incision location is covert, postoperative aesthetic effects is small.

#### Surgical Methods

**Fig. 2:**
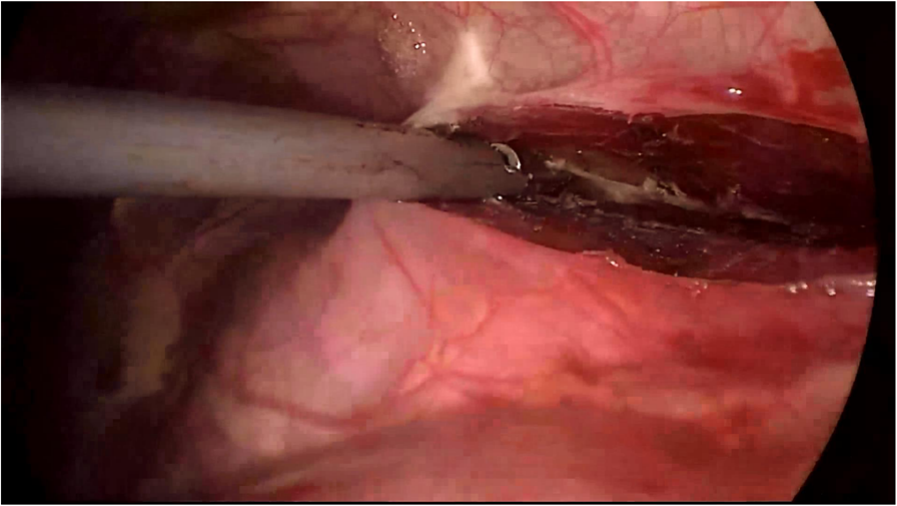
Used an Harmonic® knife or trench to open the anterior mediastinum pleura

**Fig. 3:**
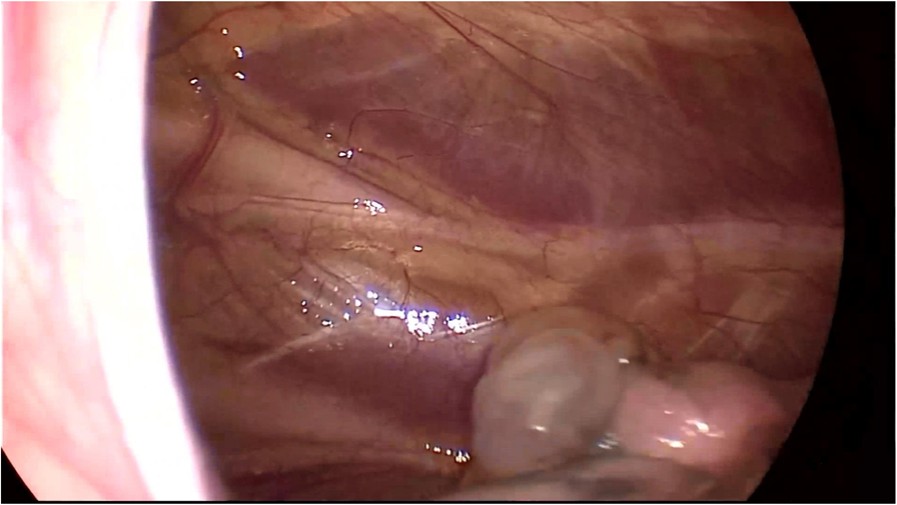
Locate the specific location of the lesion (target surgical area)in the contralateral chest

**Fig. 4:**
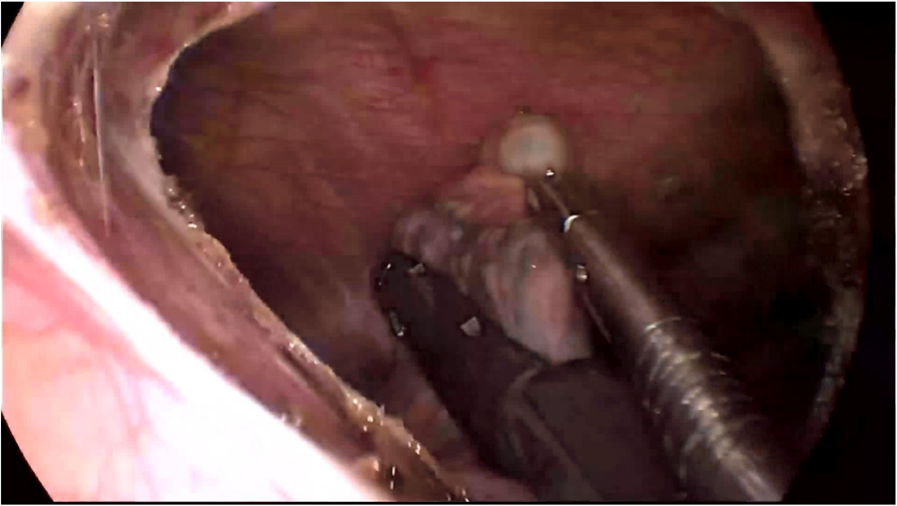
Enter into the contralateral chest via anterior mediastinum, and resect the bullae

We used 2% lidocaine to block vagal nerve, electrocoagulation or Harmonic® knife to isolate intrapleural adhesion, double joint lobule pliers to fully search the chest, in order to locate the specific location of the lesion (target surgical area). Wedge resection was used to resect the bullae (Fig. 1). When finishing operation on one side, we used an Harmonic® knife or trench to open the anterior mediastinum pleura, enter into the contralateral chest via anterior mediastinum (Fig. 2). The contralateral thoracic cavity was explored to locate the pulmonary bullae (Fig. 3), and then the pulmonary bullae was excised by a rotatable endoscopic linear stapler (Fig. 4). We avoided equipment and thoracoscopy oppressing the heart or damaging the heart. According to the situation of pulmonary bullae, we decided whether or not to carry out pleural friction. If there were multiple blebs during exploration, pleural friction should be performed to improve pleural adhesions. Pleural friction was not necessary for pulmonary bullae.

At the end of the procedure, the 28F chest tube with the side hole cut to 15 cm was inserted through the mediastinal incision into the top of the contralateral chest, aWater sealed outer end of chest tube. anesthetist to swallow the lungs with 25Kpa pressure. When the lung were fully re-expanded and no air bubbles overflow, quickly removed the chest tube while tightening the preset line and knotting. Suture subcutaneous tissue and skin.

#### Postoperative treatment

Routine ECG monitoring, observing the breath sounds using the stethoscope. According to the situation, we decided to conduct blood routine and chest ultrasound or not. Chest X-ray was performed 6 hours after sugery.

## Results

Due to mild contralateral thoracic adhesions and difficult to remove, 3 patients underwent transient bilateral single port thoracic surgery (16.7%). There was no transit thoracotomy, no death or grade 3-4 complications. The average operation time of the whole group was 44.56±17.8min. The average bleeding volume was 20±7.5ml during operation. After surgery, the average hospital stay was3. 5±1.0 days. There were no cases of postoperative catheterization. All patients were followed up for 3 months to 2 years. Two patients had their left pneumothorax recurrence (5.6%). After admission, they had thoracic closed drainage and chest CT. There were no obvious pulmonary bullae. One of them had left pneumothorax recurrence 3 months after the surgery. Pulmonary compression was about 30%. No surgical treatment was needed, thoracic puncture catheter thoracic injection was performed on this patient. The other patient had left pneumothorax recurrence 6 months after the surgery. Pulmonary compression was about 60%. We performed thoracic surgery with left thoracic perforation. During the operation, we saw a pulmonary ulcer perforation in the left lower lobe . Until the time of authoring this article, those two patients haven’t had recurrence. The rest of the patients had no recurrence or other complications. We didn’t lose any patients during follow-up time. The data of the patients are displayed in Table 1.

## Discussion

Video-assisted thoracic surgery has evolved over the years. It has become the main way to treat thoracic diseases. Pneumothorax is a common disease in thoracic surgery. Its recurrence rate is relatively high [1]. Surgical resection of the bullae is the most effective treatment for recurrent pneumothorax [2, 3]. It is reported that most pulmonary bullae is bilateral and can cause bilateral pneumothorax [4]. At the same time bilateral thoracoscopic pulmonary bullous resection is considered the best choice, which needs a longer operation time [5, 6], and increases the incidence of surgical complications.

In 2003 Migliore [7] first reported single-port video-assisted thoracic surgery (Single Port VATS, SPVATS) technology. Subsequently, the technology was gradually carried out in Europe and the United States and other countries. In 2011, Gonzalez et al [8, 9] successfully upgraded the difficulty of surgery by conducting lobectomy. In the same year, the author’s hospital [10] reported 32 patients in whom SPVATS was performed successfully with independent research and development of dual-joint surgical instruments. Then single-hole thoracoscopy was widely carried out, which has become an important means of minimally invasive thoracic surgery today.

On the other hand, scholars hope to minimize the invasion of the surgery by changing the existing anesthesia. Thus, they proposed tubeless thoracic surgery. In 2004 Pompeo et al [11] reported non-tracheal intubation in pulmonary wedge resection. In 2007 Mineo [12] reported non-tracheal intubation anesthesia in pulmonary bullous resection. In 2012 Chen Jinxing [13] reported 285 cases of lobectomy under spontaneous breathing anesthesia. They all avoided intubation airway injury and mechanical damage during intubation. Thereby they reduced the incidence of postoperative pulmonary complications. And the patients could start to eat and get out of bed earlier. They have achieved satisfactory results. With the efforts of thoracic surgeons, in 2013 He Jianxing [14, 15] completed the first Non-intubated VATS under spontaneous breathing anesthesia and completed the largest number of lobectomy in the world, followed by a more successful completion of the reconstruction process. It further proved the safety and feasibility of this type of anesthesia. Based on the above changes in anesthesia and incision optimization, the thoracic surgeon tried a "tubeless VATS" procedure, including sedation, a laryngeal mask airway to maintain spontaneous breath, vagus nerve block for simple surgical procedures such as pulmonary wedge resection and pulmonary bullous resection without chest tube. Without leaving drainage tube, they further reduced the patient's postoperative pain, no complications increased, and accelerated the rehabilitation of patients [16, 17]. In 2016 Liu Jun et al [18] reported 89 cases of spontaneous breathing anesthesia without leaving drainage tube video-assisted thoracic surgery. In 2016 Gonzalez [19, 20] and others reported three cases of "tubeless" single-port video-assisted thoracic lobectomy, and further put forward the importance of "tubeless" surgery for the development of today's thoracic surgery.

For bilateral lung disease, Kodama and Yavuzer et al. [21, 22] reported the treatment which enter the contralateral thoracic cavity from one side of the chest wall incision through the mediastinum. Wu et al. [23] first reported the successful application of unilateral VATS for bilateral pulmonary bullous resection. In 2011, Cho et al. [24] introduced a method of bilateral VATS surgery via the posterior mediastinum (between esophagus and vertebrae). In 2013, Song Nan et al [25] introduced the anterior mediastinal video-assisted thoracic treatment of bilateral pneumothorax surgery, which considered that it is better to enter through the right side of the thoracic mediastinum. Postoperative pain was lighter. The operation time was shorter. And the postoperative hospital stay was shortened. By 2015, Cho et al. [26] further compared bilateral mediastinal pulmonary bullous resection with simultaneous thoracic pulmonary bullous resection. Cho and others believed thatmediastinal bilateral bullae resection, could reduce the operation time and the patient's inconvenience, such as bilateral thoracic incision and long-term retention of the chest tube, and reduced the hospital stay.

We combined the three rapid rehabilitation surgical techniques and completed the group of 18 patients with bilateral pneumothorax. Its advantages and disadvantages are as follows:

1. Anesthesia: non-tracheal intubation anesthesia spontaneous breathing anesthesia, reduced lung inflammation and stress, avoided intubation mechanical injury, was safe and reliable. Although hypercapnia occured during surgery, it was within the acceptable range, which returned to normal soon after surgery.
2. Body position: the patients took semi-supine position. Due to gravity, the patient's heart tended to be lower and towards the back. It was easier to expose the anterior mediastinum. Due to the role of gravity and the fact that we used the double joint clamp, it was more likely to expose the contralateral lung tip and pull the left lung. Thus exploration was more comprehensive.
3. Approach: the technology of performing single port thoracic surgery from incision of the side of the lung has long been mature. There is no need to repeat. The vast majority of pneumothorax cases, pulmonary bullae are located in the tip of the lungs. It is not difficult to conduct pulmonary bullous resection by entering into the contralateral thoracic cavity through the mediastinum. However, if the lung bullae are located in the hilum and other locations, where we can not use endoscopic linear suture stapler, entering into the contralateral thoracic cavity through the mediastinum for suture, will cause surgery difficulties, if necessary, turn bilateral thoracic surgery. Therefore, the distribution of pulmonary bullae is still an important influencing factor.
  In addition, chest adhesions may have an impact on surgery. In this group of patients, three of them changed the surgical approach, due to mild contralateral thoracic adhesions. Therefore, the surgical incision needed to be evaluated by preoperative CT scan, to understand the location of the pulmonary bullae and the presence of chest adhesions. We chose to cut from the side where operation was more difficult, or the pulmonary bullae attached to the side of the chest wall.
  The anterior mediastinal approach is more suitable as a channel into the contralateral thoracic cavity, because the anterior mediastinum is more extensive than the posterior mediastinum. The following may occur, when performing thoracic surgery by entering into the contralateral thoracic cavity through mediastinum. First, for the cases where mild adhesion to the side of the chest wall, but blocked by the heart, the device can not reach the corresponding position which caused difficulties to remove pulmonary bullae. In this group of patients, two of them changed to bilateral single-hole thoracic surgery during the operation. Pulmonary bullae could not be released from the surgery side, due to contralateral chest adhesions. Second, we could not completely explore the contralateral chest. One case of recurrent cases in this group, it was confirmed that the left lower lobe of the posterior mediastinal pulmonary ulcer ruptured, by the left single-port thoracic surgery exploration.
4. The absence of chest tube significantly reduced the patient's postoperative pain, aligned with the relevant reports [17, 18]. There were no complications increased. We achieved patient’s rapid recovery and more beautiful incision; shorten the hospital stay.

Both the tubeless-SPVATS via anterior mediastinal approach and the subxiphoid operation are unilateral single-port thoracoscopic surgery, and have the defects of incomplete exploration and cardiac compression. Subxiphoid operation minimally interferes with the intercostal nerve, and the postoperative pain may be milder than this surgical method. In the subxiphoid operation, there is incomplete exploration during exploring the pulmonary dorsal in the bilateral chest. Meanwhile, because the operation path is long, the operation is difficult under a single incision. In addition, subxiphoid operation may cause cardiac compression and arrhythmia, and it will be affected by the heart's pulsation. By contrast, the tubeless-SPVATS via anterior mediastinal approach can well explore the affected side, thus ensuring the therapeutic efficacy in the affected side. Nevertheless, there is possibility of incomplete exploration of the contralateral thoracic cavity. As a result, the pneumothorax recurrence rate of the contralateral thoracic cavity may be higher compared to the chest side of incision. This potential problem can be solved by using flexible thoracoscope and flexible instrument, which can effectively prevent the mutual interference between the thoracoscope and instrument during surgery. Meanwhile, using a flexible Olympus electronic thoracoscope can provide a wider and more comprehensive surgical view.

We believe that this approach is particularly suitable for the cases with pulmonary bullae concentrated in the tip of the lungs. The overall efficiency of single incision to solve the bilateral pneumothorax problem is satisfactory. This tubeless-SPVATS surgical method combined the advantages of spontaneous breathing under general anesthesia, tubeless, single-port and transthoracic mediastinal approach, all of which are help to on reduce postoperative discomfort and promote recovery. The patients can eat normally at 6 hours after surgery and had no postoperative hoarseness or sore throat. Compared to the conventional bilateral approach surgery by Lang-Lazdunski et al. [27], the tubeless-SPVATS had fewer incisions, reduced surgery operative time (44.56±17.8 vs. 168±17 min) and postoperative hospital stay (3.5±1.0 vs. 7.7±1.4 days). The postoperative complication in this study was pneumothorax recurrence (11.1%), while those in Lang-Lazdunski et al.’s study includes prolonged air leak (16.5%), incomplete lung reexpansion (25%), and pleural effusion (8.5%). These comparisons seem to suggest the beneficial effect of the tubeless-SPVATS. However, this study is limited by the relatively small sample size, and a prospective randomized controlled study with a large sample size should be conducted to further validate the the therapeutic efficacy and outcome of the tubeless-SPVATS.

In short, rapid rehabilitation surgery is the future direction of development. With the deepening of the study, the improvement of surgical instruments and surgical approach improvements, it will further promote the advance of thoracic surgery no matter what kind of incision and surgical approach.

## Conclusions

Tubeless-SPVATS via anterior mediastinum is a safe and feasible treatment for patients with simultaneous bilateral PSP or pulmonary bullae. However,contralateral thoracic is not explored fully enough. And when contralateral lung bullae are located near the hilum, endoscopic linear stapler cannot be ceasily used to conduct suture. Thus, the recurrence rate after performing Tubeless-SPVATS may be increased compared to performing thoracotomy. However, compared to bilateral thoracic surgery, this method reduced postoperative pain. And it took significantly less time than bilateral thoracic surgery. Thus, this method has some clinic value.

## Abbreviations

PSP: Primary spontaneous pneumothorax
SPVATS: Single-port video-assisted thoracic surgery
